# Vitrification affects the expression of matrix metalloproteinases and their tissue inhibitors of mouse ovarian tissue

**Published:** 2016-03

**Authors:** Reza Asadzadeh, Shima Khosravi, Saeed Zavareh, Mohammad Taghi Ghorbanian, Seyed Hassan Paylakhi, Seyed Reza Mohebbi

**Affiliations:** 1 *School of Biology, Damghan University, Damghan, Iran.*; 2 *Institute of Biological Sciences, Damghan University, Damghan, Iran.*

**Keywords:** *Vitrification*, *Pre antral follicles*, *Matrix metalloproteinases*, *Mouse*

## Abstract

**Background:**

One of the most major obstacles of ovarian tissue vitrification is suboptimal developmental competence of follicles. Matrix metalloproteinases 2 (MMP-2) and 9 (MMP-9) and their tissue inhibitors TIMP-1 and TIMP-2 are involved in the remodeling of the extracellular matrix in the ovaries.

**Objective:**

This study aimed to evaluate the expression of MMP-2, MMP-9, TIMP-1, and TIMP-2 genes in the preantral follicles derived from vitrified mouse ovaries.

**Materials and Methods:**

In this experimental study, the gene expression of MMP-2, MMP-9, TIMP-1, and TIMP-2 in the isolated preantral follicles derived from fresh and vitrified ovaries of 14-16 days old female mice through real time qRT-PCR was evaluated. Developmental parameters, including survival rate, growth, antrum formation and metaphase II oocytes were also analyzed.

**Results:**

The developmental parameters of fresh preantral follicles were significantly higher than vitrified preantral follicles. The TIMP-1 and MMP-9 expression levels showed no differences between fresh and vitrified preantral follicles (p=0.22, p=0.11 respectively). By contrast, TIMP-2 expression significantly decreased (p=0.00) and MMP-2 expression increased significantly (p=0.00) in vitrified preantral follicles compared with to fresh ones.

**Conclusion:**

Changes in expression of MMP-2 and TIMP-2 after ovarian tissues vitrification is partially correlated with decrease in follicle development.

## Introduction

Ovarian tissue vitrification provides an opportunity for women who undergo chemotherapy and radiotherapy to preserve their fertility (1). The vitrification of ovarian tissue is followed by several changes that affect subsequent ovarian follicular development (2). Follicular development starts in primordial follicle stage, granulosa cells proliferate, theca cell layer differentiates from ovarian stroma and basement membrane is deposited between theca cells and granulosa cells (1). This broad follicular growth requires a consistent and extensive remodeling of granulosa cells, basement membrane, and extra cellular matrix (ECM) of ovarian follicles (3). 

Matrix metalloproteinases (MMPs) and their inhibitors (tissue inhibitors of metalloproteinases, TIMPs) play crucial roles in remodeling of ovarian ECM (4-7). The MMP activity is stimulated by follicular development-related events. Expression pattern and position of MMP genes are consistent with changes in follicular theca and granulosa layer (5). It has been shown that, MMPs directly regulate the growth and development of ovarian follicles by remodeling ECM, MMPs also indirectly regulate these processes by activating or interacting with growth factors and cytokines (8-10). Gelatinase A (MMP-2) and gelatinase B (MMP-9) digest denatured collagens and gelatins (1, 11). 

Gelatinases bind to heparan sulfate on cell surface to control tissue remodeling, matrix-cell reaction, and local cytokine degeneration (12). Gelatinases may also regulate critical messages that affect cell proliferation. Furthermore, gelatinases may control the activity of growth factors through the binding and breakdown of proteins (13). Imai *et al* showed that MMP-2 is a marker of healthy ovarian follicles (14). The MMP-9 secretion is related to the accuracy of follicular development (8).

The MMP activity is controlled by inhibitors in an extracellular environment. TIMPs include TIMP-1, TIMP-2, TIMP-3, and TIMP-4. These inhibitors are found in reproductive tissues and are locally expressed. TIMPs regulate a number of aspects of the ovulatory process, such as ECM remodeling, cell growth, and steroid hormone production (15). TIMP-1 and TIMP-2 are glycoproteins that bind to MMPs and inhibit their activated forms (16). TIMP-2 likely binds to MMP-2, whereas TIMP-1 preferentially binds to MMP-9, as such, the gelatinase activity should be regulated because this activity in turn controls homeostasis of basement membrane. Furthermore, ovarian TIMPs may act independently from their inhibitory capacities. TIMPs also function as paracrine and endocrine factors in cell proliferation and differentiation, steroid hormone production, and follicular proteolysis regulation during ovarian follicular development (8, 15, 16).

The MMPs system and their inhibitors regulate the normal maturation and atresia of ovarian follicles to achieve numerous competent follicles (8). The activities of MMP-2, MMP-9, TIMP-1, and TIMP-2 can be considered as a marker of the accuracy of follicular development (9). Thus, the evaluation of these variables may explain the poor results of ovarian tissue vitrification. Hence, the present study aimed to investigate the expression levels of the MMP-2, MMP-9, TIMP-1, and TIMP-2 genes of the preantral follicles derived from vitrified mouse ovaries.

## Materials and methods

**Reagents**

Alpha minimum essential medium (α- MEM), Dulbecco’s phosphate- buffered saline (DPBS), and fetal bovine serum (FBS) were purchased from GIBCO (UK). Other reagents were obtained from Sigma-Aldrich (UK) unless otherwise mentioned. Culture media were prepared with deionized water (Milli-Q).

**Animals**

In this experimental animal study, 14-16 days old female Naval Medical Research Institute (NMRI) mice (n=3 for each group) were obtained from Pasteur Institute of Iran (Karaj, Iran) and transferred to Animal House of Damghan University. The mice were kept under standard conditions of 12 hr light/dark cycle at 24^o^C with food and water ad libitum in accordance with guideline of Institutional Animal Care and Use Committee at Damghan University. 

The mice were sacrificed through cervical dislocation. Ovaries were removed and placed immediately in 6 cm Petri dishes containing drops of α-MEM supplemented with 10% FBS, 100 IU/ml penicillin, and 75 µg/ml streptomycin buffered with 25 mM 4-(2-hydroxyethyl)-1-piperazineethanesulfonic acid (HEPES) and 2.2 g/l sodium bicarbonate covered with mineral oil. The ovaries were stored in an incubator (Memmert, Germany) for 30 min at 37^o^C with 5% CO_2_ and 98% moisture to adapt to the new condition. Afterward, the redundant tissues surrounding the ovaries were removed using a 29-gauge needle attached to an insulin syringe under a stereomicroscope (Nikon, Japan). The ovaries were randomly divided into two groups, namely, vitrified and fresh ovaries.

**Experimental design**

This study was performed in two steps. First, it was evaluated the developmental parameters, including the rates of survival, growth, antral formation, and metaphase II oocytes of the preantral follicles derived from fresh and vitrified ovaries. Second, the expression levels of the MMP-9, MMP-2, TIMP-1 and TIMP-2 genes were investigated in both groups.

**Vitrification and warming of ovarian tissues**

The ovarian tissue was vitrified in accordance with previously described methods with some modifications (17). In brief, the ovaries were placed in an equilibrium solution containing 7.5% (v/v) dimethyl sulfoxide (DMSO) and 7.5% (v/v) ethylene glycol (EG) in the DPBS medium for 10 min. Afterward, the ovaries were placed in a vitrification solution containing 15% EG (V/V), 15% DMSO (v/v), 20% FBS, and 1 M sucrose for 2 min. The ovarian tissue was transferred immediately to the tip of a Cryolock™ and immersed in liquid nitrogen. The ovaries were stored in liquid nitrogen for at least one week. Subsequently, the tip of the Cryolock™ was immediately placed in warming solutions (1, 0.5, and 0.25 M sucrose in DPBS at 5 min intervals). The ovaries were washed with several drops of α-MEM containing 10% FBS to remove the warming solution and transferred to the α- MEM culture medium supplemented with 10 FBS, 2.2 g/l sodium bicarbonate, 25 mM HEPES, 100 IU/ml penicillin, and 75 µg/ml streptomycin.

**Preantral follicular isolation**

The preantral follicles of the fresh and vitrified ovaries were mechanically isolated with a 29-gauge needle attached to an insulin syringe under a stereomicroscope (Nikon, Japan). The preantral follicles with a diameter of approximately 140-160 μm with a central oocyte and two to three layers of granulosa and theca cells were considered healthy. the healthy preantral follicles were selected and cultured in vitro.

**In Vitro culture of preantral follicle**

The preantral follicles were cultured in 25 µl of α-MEM containing 5% FBS, 0.1 IU/ml recombinant human follicle-stimulating hormone, 1% insulin, transferrin, and selenium (ITS), 10 ng/ml epithelial growth factor, 2.2 g/l sodium bicarbonate, 100 IU/ml penicillin, and 75 µg/ml streptomycin in 6 cm Petri dishes for 12 days. At least half of the culture medium was replaced with a new culture medium every other day. The diameters of the preantral follicles were determined by calculating the average of two perpendicular diameters of each follicle on the second and fourth days of the cultivation period. 

The survival rates of the preantral follicles were examined on 2, 4, 6, 8, 10, and 12 days. The dark preantral follicles without egg, but exhibiting diminutive growth were considered as degenerate follicles. The transparent areas in the granulosa cell mass around the oocyte were considered as the antral-like cavity. Ovulation was induced by changing the culture medium with a fresh medium containing 1.5 IU/ml human chorionic gonadotropin on the 12^th^ day. The developmental stages of the released oocytes (metaphase I [MI] and MII) were investigated after 24-48 hr.

**Reverse transcription and quantitative PCR (qRT-PCR)**

The total RNA extraction of the preantral follicle was performed on the first day of the culture period using the RNXTM- Plus kit (Cinnagen, Tehran, Iran) in accordance with the manufacturer’s instructions. RNA preparations were treated with DNase to eliminate any DNA contamination. The quality of RNA was assessed using the density ratio of 28S-18S rRNA bands. The first-strand cDNA was synthesized from 1,000 ng of the total RNA by using avian myeloblastosis virus reverse transcriptase (Cinnagen, Tehran, Iran) primed by random hexamers in accordance with the manufacturer’s instructions.

Real- time RT-PCR was performed using a Rotor- Gene 6000 machine (Corbett, USA) in a QuantiFast SYBR Green PCR kit (Qiagen, Germantown, MD, USA). DNA was initially denatured for 10 min at 94^o^C and then amplified for 40 cycles of 15 Sec at 94^o^C, 25 Sec at 60^o^C, and 30 Sec at 72^o^C. The specificity of the PCR products was confirmed through melting curve analysis and agarose gel electrophoresis. β-actine was used as a control gene in real-time qRT-PCR. The detailed information of the primers is provided in table I. PCR efficiency of each gene was determined on the basis of the standard curves. Relative quantification analysis was performed using the 2-ΔΔCT method with the Rotor-Gene 6000 Series version 1.7 (18).

**Statistical analysis**

Statistical analysis was performed using SPSS version 16 software package (SPSS Inc., Chicago, IL, USA). Data were statistically analyzed using Student’s independent t-test. A significance level of p<0.05 was considered. Each experiment was carried out with at least three replicates. Data were expressed as means±SD.

**Table I:** List of used primers

**Primer**	**Sequence**	**Primer Size**	**Product size (base pairs)**	**Tm (** ^o^ **C)**
ACTIN-B-F	5-GATTACTGCTCTGGCTCCTAG-3	21	147	54.51
ACTIN-B-R	5-GACTCATCGTACTCCTGCTTG-3	21		56.02
MMP-2-F	5-GCCCCGAGACCGCTATGTCCACT-3	23	170	70.11
MMP-2-R	5-GCCCCACTTCCGGTCATCATCGTA-3	24		70.52
MMP-9-F	5-GCGCCACCACAGCCAACTATG-3	21	379	66.66
MMP-9-R	5-TGGATGCCGTCTATGTCGTCTTTA-3	24		63.55
TIMP-1-F	5-ACTCGGACCTGGTCATAAGGGC-3	22	466	64.37
TIMP-1-R	5-TTCCGTGGCAGGCAAGCAAAGT-3	22		68.75
TIMP-2-F	5-GGCAACCCCATCAAGAGGA-3	19	167	61.77
TIMP-2-R	5-CCTTCTGCCTTTCCTGCAATTAG-3	23		62.09

## Results

**Growth and development**

**Figure1.**
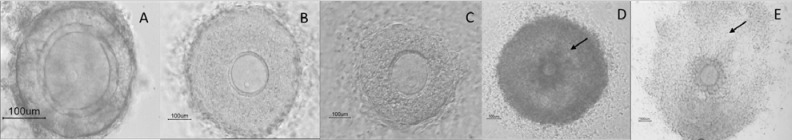
Images of the in vitro cultured mouse pre-antral follicles Pre-antral follicle on initial day (A), day 4 (B), day 6 (C), day 8 (D) and day 10 (E) Antral-like cavities are indicated by black arrow

The mean diameter and the rates of developmental parameters of the vitrified and fresh preantral follicles are summarized in tables II, III. From the second day onwards, the preantral follicles initially became attached to the culture dish through granulosa and theca cell proliferation. After the fourth day, the preantral follicles formed an irregular and diffused appearance such that their diameters could not be easily measured (Figure 1).

In the first phase, the mean diameters of the preantral follicles of the groups showed no significant change on the first day of culture (t-value (t) (543.05)=1.09, p=0.057). By contrast, the diameters of the fresh preantral follicles on the second (t(565.27)=17.66, p=0.00) and fourth days (t(482.44)=98.52, p=0.00) of culture were significantly higher than those of the preantral follicles isolated from vitrified ovaries. The survival (t(5.58)=7.14, p=0.001), antrum formation (t(4.82)=12.21, p=0.00), and ovulation (t(4.49)=6.156, p=0.002) rates of the preantral follicles derived from vitrified ovaries were significantly lower than those of the fresh preantral follicles. 

Moreover, the rate of the MII oocytes produced from the fresh preantral follicles was significantly higher than those of the preantral follicles derived from vitrified ovaries (t(4.06)=5.26, p=0.006). mRNA expression of MMP-2, MMP-9 and TIMP 1-2 compared with the MMP-2 gene expression in the control group, the MMP-2 gene expression in the preantral follicles derived from vitrified group significantly increased (Figure 1, t(3)=28.77, p=0.00). In addition, the MMP-9 gene expression decreased slightly, but not significantly in the vitrified preantral follicles compared with the fresh preantral follicles (t(3)=-5.71, p=0.11). 

**Figure 2:**
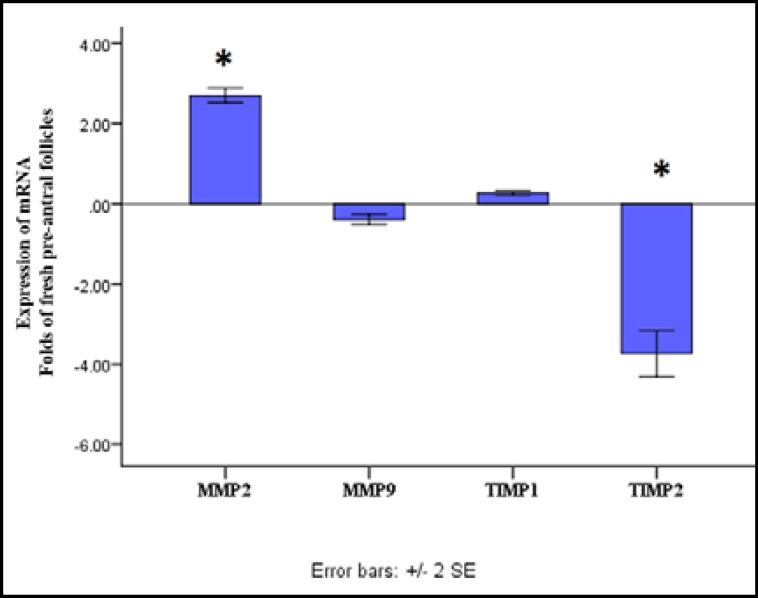
Expression levels of MMP2, MMP9, TIMP1 and TIMP2 mRNA of the vitrified preantral follicle in comparison with those of fresh pre-antral follicles. *: Indicate significant difference in comparison with fresh preantral follicles (p<0.05).

In comparison with the control group, the TIMP-2 gene expression in the preantral follicles derived from vitrified group significantly decreased (Figure 2, t(3)=12.94, p=0.00). By contrast, the TIMP-1 gene expression did not show significant differences between the preantral follicles derived from vitrified ovaries and the fresh preantral follicles (Figure 2, t(3)=10.52, p=0.22).

**Table II.** Diameter of preantral follicles derived from fresh and vitrified ovaries during the cultivation period

**Group**	**NO of preantral follicle**	**InitialTime**	**Day2**	**Day4**
Fresh	314	149.54 ± 5.85	249.57 ± 16.83*	441.12 ± 11.14*
Vitrified	256	150.49 ± 5.91	226.22 ± 14.7	370.12 ± 5.64

*: indicate a significant difference in the same column (p<0.05)

**Table III:** Rates of developmental parameters of cultured preantral follicles derived from fresh and vitrified ovaries

**Groups**	**No of preantral follicle**	**Survival**	**Degenerated**	**Antrum**	**Ovulation**	**MII**	**MI**	**GV**
Fresh	314	298 (94.99 ± 1.44)*	16 (5.01 ± 1.43)*	279 (88.93 ± 1.4)	246 (78.67 ± 6.06)*	193 (62.19 ± 8.15)*	33 (10.35 ± 3.08)	20 (6.13 ± 2.30)
Vitrified	256	221 (86.46 ± 1.91)	35 (13.54 ±1.91)	185 (72.18 ± 2.36)	148 (57.67 ± 3.13)	100 (38.86 ± 3.48)	30 (11.62 ± 1.76)	18 (7.18 ± 1.77)

Data are presented as n (%±SD)

GV: Germinal Vesicle

MI: metaphase I

MII: metaphase II.

*: indicate a significant difference in the same column (P<0.05)

## Discussion

The growth, survival, antrum formation, and ovulation rates of the fresh preantral follicles were significantly higher than those of the vitrified preantral follicles. Two types of serious injuries, namely, crystallization and toxicity to high cryoprotectant concentrations, may occur during cryopreservation. In vitrification methods, ice crystal formation is largely eliminated, but other injuries, such as chemical toxicity and osmotic injuries, likely occur (19). Our results are consistent with those in previous studies; the development rates of the vitrified preantral follicles compared with those of the fresh preantral follicles, significantly decreased (20-22). 

This finding has been attributed to ultrastructural cellular changes, such as nuclear fragmentation, mitochondrial breakdown, cell membrane wrinkling, and cytoplasmic vacuole formation (23, 24). During vitrification, numerous chemical and physical conditions cause changes in membrane properties and lipid peroxidation; these changes destroy cellular integrity and cause an increased production of reactive oxygen species; leading to delay in follicle development (25-27). The number of granulosa cells decreases in the follicles of vitrified ovaries (21). 

These granulosa cells regulate the growth, antrum formation, and ovulation of preantral follicles by secreting growth factors. Therefore, the low rates of the growth, survival, and antrum formation of the vitrified group may be partly caused by the decrease in the secretion of growth factors by the granulosa cells in the vitrified group. This finding is consistent with that of Cortvrindt *et al,* who found that the concentration of growth factors in vitrified follicles could be reduced (28). 

The reconstruction of the basement membrane of granulosa and theca cells during ovarian follicle development is partly dependent on the MMP activity (8, 9). The basement membrane plays a critical role in the differentiation, proliferation, survival, and migration of cells during the embryonic development of ovary; the basement membrane also functions as a selective barrier and structural framework of ovaries and follicles during puberty (29). MMPs may directly regulate the remodeling of ovarian tissues and follicles and indirectly stimulate follicular growth through the regulation of growth factor secretion and cytokine activation (10). Hence, MMPs can stimulate cell proliferation through interaction with cytokines (8).

Considering the roles of MMPs, it can be concluded that the changes in the growth factor secretion of granulosa cells and those in the cell membrane are related to the changes in the MMP expression following vitrification. This finding is consistent with that in our study, which showed that the TIMP-2 expression decreased and the MMP-2 expression increased in the vitrified preantral follicles compared with the fresh preantral follicles. Cryoprotectants damage the basement membrane by eradicating collagen type IV during vitrification; in turn, follicular development is impeded (30). MMP-2 is involved in the disruption of collagen type IV, thereby causing damage to the basement membrane (31). 

Therefore, it seems that the increased MMP-2 activity associated with disruption of collagen type IV followed by using cryoprotectant possibly results in the reduction of vitrified follicular development. This observation is consistent with this study, which showed that the MMP-2 gene expression increased after vitrification. Kim *et al *showed that the collagen type IV expression in embryonic stem cells decreased after these stem cells were cryopreserved. They then injected collagen type IV and laminin to the vitrified cells and observed that the survival rate and cell morphological characteristics improve after the cells are vitrified (32). 

These findings confirm the impairment of the preantral follicles through the destruction of collagen type IV by increasing the MMP-2 activity. Bagavandoss *et al* also observed that follicular atresia following hypophysectomy is associated with an increase in MMP-2 and MMP-9 activities (4). This finding is in agreement with the results of the present study, which showed that the increase in MMP-2 activity was associated with the increase in degeneration rate and decrease in the development rate in the vitrified preantral follicles. The disassembly of the follicle structure that resulted from the increased MMP-2 expression leads to an increase in vitrified follicle degeneration. TIMP-2 exhibits high affinity to MMP-2, whereas TIMP-1 preferentially binds to MMP-9. Thus, a change in the MMP-2 gene expression causes a change in the TIMP-2 gene expression (8, 9). 

These findings confirm our results, which showed that the TIMP-2 expression in the isolated preantral follicles derived from vitrified ovaries was significantly lower than that of the fresh preantral follicles; conversely, the MMP-2 gene expression increased. Any change in TIMP-1 expression levels is followed by a change in MMP-9 expression levels because TIMP-1 preferentially controls MMP-9 (8, 9). This finding is in agreement with that observed in our study, which revealed that the MMP-9 and TIMP-1 expression did not significantly change between vitrified and control preantral follicles. Ovarian TIMPs not only play roles in uterine and ovarian matrix remodeling but also likely act independently as autocrine factors in cell proliferation, cell differentiation, and steroidogenesis during follicular development (8, 9). 

As a result, the decreased TIMP-2 gene expression partly supports the reduction of development of vitrified follicles. Although MMPs and TIMPs are implicated in follicular growth and development, this study is the first to demonstrate that the changes in MMP-2 and TIMP-1 expression are correlated with the decrease in growth and follicle development after vitrification of ovarian tissues are vitrified.

## References

[1] Visse R, Nagase H (2003). Matrix metalloproteinases and tissue inhibitors of metalloproteinases: structure, function, and biochemistry. Circul Res.

[2] Salehnia M, Abbasian Moghadam E, Rezazadeh Velojerdi M (2002). Ultrastructure of follicles after vitrification of mouse ovarian tissue. Fertil Steril.

[3] Balbin M, Fueyo A, Lopez JM, Diez-Itza I, Velasco G, Lopez-Otin C (1996). Expression of collagenase-3 in the rat ovary during the ovulatory process. J Endocrinol.

[4] Bagavandoss P (1998). Differential distribution of gelatinases and tissue inhibitor of metalloproteinase-1 in the rat ovary. J Endocrinol.

[5] Goldman S, Shalev E (2004). MMPS and TIMPS in ovarian physiology and pathophysiology. Front Biosci.

[6] Smith M, McIntush E, Ricke W, Kojima F, Smith G (1999). Regulation of ovarian extracellular matrix remodelling by metalloproteinases and their tissue inhibitors: effects on follicular development, ovulation and luteal function. J Reprod Fertil.

[7] Ny T, Wahlberg P, Brändström IJ (2002). Matrix remodeling in the ovary: regulation and functional role of the plasminogen activator and matrix metalloproteinase systems. Mol Cell Endocrinol.

[8] Curry TE Jr, Osteen KG (2003). The matrix metalloproteinase system: changes, regulation, and impact throughout the ovarian and uterine reproductive cycle. Endocrine Rev.

[9] Curry TE Jr, Osteen KG (2001). Cyclic changes in the matrix metalloproteinase system in the ovary and uterus. Biol Reprod.

[10] Edwards DR, Beaudry PP, Laing TD, Kowal V, Leco KJ, Leco PA (1996). The roles of tissue inhibitors of metalloproteinases in tissue remodelling and cell growth. Int JObes.

[11] Hagglund AC, Ny A, Leonardsson G, Ny T (1999). Regulation and localization of matrix metalloproteinases and tissue inhibitors of metalloproteinases in the mouse ovary during gonadotropin-induced ovulation. Endocrinology.

[12] Yu WH, Woessner JF Jr (2000). Heparan sulfate proteoglycans as extracellular docking molecules for matrilysin (matrix metalloproteinase 7). JBiol Chem.

[13] Robinson LL, Sznajder NA, Riley SC, Anderson RA (2001). Matrix metalloproteinases and tissue inhibitors of metalloproteinases in human fetal testis and ovary. Mol Hum Reprod.

[14] Imai K, Khandoker MA, Yonai M, Takahashi T, Sato T, Ito A (2003). Matrix metalloproteinases-2 and -9 activities in bovine follicular fluid of different-sized follicles: relationship to intra-follicular inhibin and steroid concentrations. Domest Animal Endocrinol.

[15] Brew K, Dinakarpandian D, Nagase H (2000). Tissue inhibitors of metalloproteinases: evolution, structure and function. Biochem Biophys Acta.

[16] Gomez DE, Alonso DF, Yoshiji H, Thorgeirsson UP (1997). Tissue inhibitors of metalloproteinases: structure, regulation and biological functions. Eur J Cell Biol.

[17] Chen S-U, Chien C-L, Wu M-Y, Chen T-H, Lai S-M, Lin C-W (2006). Novel direct cover vitrification for cryopreservation of ovarian tissues increases follicle viability and pregnancy capability in mice. Hum Reprod.

[18] Schmittgen TD, Livak KJ (2008). Analyzing real-time PCR data by the comparative CT method. Nat Prot.

[19] Vajta G, Kuwayama M (2006). Improving cryopreservation systems. Theriogenology.

[20] Choi J, Lee J-y, Lee E, Yoon B-K, Bae D, Choi D (2007). Cryopreservation of the mouse ovary inhibits the onset of primordial follicle development. Cryobiology.

[21] Newton H, Illingworth P (2001). In-vitro growth of murine pre-antral follicles after isolation from cryopreserved ovarian tissue. Hum Reprod.

[22] Newton H (1998). The cryopreservation of ovarian tissue as a strategy for preserving the fertility of cancer patients. Hum Reprod Update.

[23] Abdollahi M, Salehnia M, Salehpour S, Ghorbanmehr N (2013). Human ovarian tissue vitrification/warming has minor effect on the expression of apoptosis-related genes. Iran Biomed J.

[24] Hernandez-Barrantes S, Toth M, Bernardo MM, Yurkova M, Gervasi DC, Raz Y (2000). Binding of active (57 kDa) membrane type 1-matrix metalloproteinase (MT1-MMP) to tissue inhibitor of metalloproteinase (TIMP)-2 regulates MT1-MMP processing and pro-MMP-2 activation. JBiol Chem.

[25] Hatami S, Zavareh S, Salehnia M, Lashkarbolouki T, Ghorbanian MT, Karimi I (2014). Total oxidative status of mouse vitrified pre-antral follicles with pre-treatment of alpha lipoic acid. Iran Biomed J.

[26] Hatami S, Zavareh S, Salehnia M, Lashkarbolouki T, Karimi I (2014). Comparison of oxidative status of mouse pre-antral follicles derived from vitrified whole ovarian tissue and vitrified pre-antral follicles in the presence of alpha lipoic acid. J Obstet Gynaecol Res.

[27] Rahimi G, Isachenko E, Sauer H, Isachenko V, Wartenberg M, Hescheler J (2003). Effect of different vitrification protocols for human ovarian tissue on reactive oxygen species and apoptosis. Reprod Fertil Dev.

[28] Cortvrindt R, Smitz J, Van Steirteghem AC (1996). A morphological and functional study of the effect of slow freezing followed by complete in-vitro maturation of primary mouse ovarian follicles. Hum Reprod.

[29] Poschl E, Schlotzer-Schrehardt U, Brachvogel B, Saito K, Ninomiya Y, Mayer U (2004). Collagen IV is essential for basement membrane stability but dispensable for initiation of its assembly during early development. Development.

[30] Luvoni GC, Tessaro I, Apparicio M, Ruggeri E, Luciano AM, Modina SC (2012). Effect of vitrification of feline ovarian cortex on follicular and oocyte quality and competence. Reprod Domest Anim.

[31] Okamoto T, Niu R, Yamada S (2003). Increased expression of tissue inhibitor of metalloproteinase-2 in clear cell carcinoma of the ovary. Mol Hum Reprod.

[32] Kim SJ, Park JH, Lee JE, Kim JM, Lee JB, Moon SY (2004). Effects of type IV collagen and laminin on the cryopreservation of human embryonic stem cells. Stem Cells.

